# Poor sleep quality and erectile dysfunction in students from a Peruvian University: A cross-sectional study

**DOI:** 10.3389/fpubh.2023.932718

**Published:** 2023-02-01

**Authors:** Pierina Gutierrez-Velarde, Mario J. Valladares-Garrido, C. Ichiro Peralta, Victor J. Vera-Ponce, J. Antonio Grandez-Urbina

**Affiliations:** ^1^School of Medicine, Universidad Ricardo Palma, Lima, Peru; ^2^South American Center for Education and Research in Public Health, Universidad Norbert Wiener, Lima, Peru; ^3^Epidemiology Office, Hospital Regional Lambayeque, Chiclayo, Peru; ^4^School of Medicine, Universidad Nacional Federico Villarreal, Lima, Peru; ^5^Facultad de Medicina, Instituto de Investigación en Ciencias Biomédicas de la Universidad Ricardo Palma, Lima, Peru; ^6^Universidad Tecnológica del Perú, Lima, Peru; ^7^Universidad Continental, Lima, Peru

**Keywords:** erectile dysfunction, sleep disorders, International Index of Erectile Function, Pittsburgh Sleep Quality Index, Berlin questionnaire

## Abstract

**Objective:**

We aimed to evaluate the association between sleep quality and erectile dysfunction in young university students.

**Methods:**

A cross-sectional survey was conducted in men aged 18–30 years from Universidad Ricardo Palma, Lima, Peru. The survey comprised the International Index of Erectile Function, Pittsburgh Sleep Quality Index, Berlin questionnaire, and questions related to sociodemographic data. Prevalence ratios were estimated with generalized linear models.

**Results:**

Of 381 participants, the median age was 23 years. Half of the students (50.9%) had poor sleep quality, of which 72.7% had mild erectile dysfunction and 20.6% mild to moderate dysfunction. Prevalence of erectile dysfunction was significantly higher in students with poor sleep quality than in students with good sleep quality (aPR = 6.48; 95% CI: 4.58–9.17) after adjusting for age, academic year, nutritional status, and sleep apnea. In a subsequent exploratory analysis, sleep apnea was associated with a higher prevalence of erectile dysfunction (aPR = 1.19; 95% CI: 1.01–1.39), while overweight (aPR = 0.85; 95% CI: 0.76–0.95) and obesity (aPR = 0.65; 95% CI: 0.52–0.82) were associated with a lower prevalence of this condition.

**Conclusion:**

Poor sleep quality was independently associated with erectile dysfunction in young university students. This finding suggests that male students are at risk for sexual problems due to possible academic demands and relationship issues.

## Introduction

Erectile dysfunction (ED) is a common problem among men that affects the quality of life of themselves and their partners. Two important cohort studies helped to understand the frequency of ED. The Massachusetts Male Aging Study reported an overall prevalence of ED of 52% (1), while the European Male Aging Study showed that 30% of men experienced ED. A more recent international study reported a prevalence between 37.2 and 48.6% (2). In Latin America, ED was found in 53.4% of men older than 40 years (3). Despite the growing literature on ED, the incidence of ED is often underestimated, and the epidemiology of this condition is commonly neglected among young men.

ED among young men is considered to be mainly triggered by psychological burden (4). Academic life is an important stage in young's life, and this can lead to high levels of distress, affecting their quality of life and academic performance. Some studies have shown that mental disorders affect health dimensions such as physical, social, and environmental. However, few studies have explored how mental disorders can affect the sexual function (5).

Sleep quality is an essential aspect of physical and mental health (6). It promotes well-being and prevents different conditions from depression to cardiovascular disorders (7).

Poor sleep quality can affect multiple and relevant processes. On the physical aspects, it may cause diabetes, obesity, cardiovascular diseases, and even mortality (8). On the psychological aspects, it may lead to attention deficit, cognitive disability, and depression (9, 10). Therefore, sleep quality is an important aspect in health.

The relationship between sleep quality and ED has been described in an increasing number of studies. It has been shown that ED can be caused by several sleep disorders, such as sleep obstructive apnea, insomnia, and nocturia (6). It has also been reported that men with any comorbidity had 1.79-fold the risk of ED compared with healthy men, but the risk was up to 3.34-fold with the inclusion of sleep disorders (11). Poor sleep quality may alter testosterone and oxygen levels, with localized endothelial dysfunction (12). However, most studies have focused on middle-aged and older adults. Among the few reports in young men, one showed that 47% of medical students with sleep disorders experienced ED (13). As sleep is an important aspect in young's lives, there is a need to better understand the influence of sleep disturbance on ED.

Therefore, we aimed to evaluate the association between poor sleep quality and ED in young university students. We stated three research questions: (1) What is the prevalence of ED in university students? (2) By which extent does the prevalence of ED vary according to the students' characteristics? (3) Does the presence of poor sleep quality influence on the development of ED? For the purpose of this study, three hypotheses were stated: (1) The prevalence of ED is high in the study group; (2) There are some common characteristics among young students that influence the development of ED, such as early academic years, obesity, sleep apnea, and poor sleep quality; and (3) poor sleep quality is independently associated with the development of ED.

## Materials and methods

### Study design, population, and sample

A cross-sectional survey was conducted in students aged 18–30 years at the Ricardo Palma University (URP) during the 2018 academic year. The URP is a private university located in Lima, Peru. It provides undergraduate education at eight faculties, of which four were included for data collection: medicine, architecture, modern linguistics, and engineering.

Inclusion criteria were students who agreed to participate in the study and completed all the variables of interest. Exclusion criteria were students reporting any neurological disorder, anatomical alteration of the penis, and no frequent sexual activity in the last 6 months.

Based on a 99% confidence interval, a statistical power of 90 and an expected prevalence rate of 1.5, a sample size of 368 students was calculated. A non-probability sampling method was applied through face-to-face interviews with students on campus.

### Measures

Erectile dysfunction was measured with the International Index of Erectile Function (IIEF-5) scale. The IIEF-5 scale was designed by Rosen et al. (14) and has five questions assessing erectile function, orgasmic function, sexual appetite, sexual satisfaction, and general satisfaction. The instrument has been validated in a sample of 75 Peruvian patients aged from 18 to 60 years at a referral hospital in Lima, Peru, showing content, criteria, discriminatory, and divergent validity, and presenting good internal consistency (Cronbach's alpha >0.8 in the five domains) (15). Scores range from 5 to 25 and classify ED into five categories: severe ED (5–7 points), moderate ED (8–11 points), mild to moderate ED (12–16 points), mild ED (17–21 points), and no ED (22–25 points) (16). For regression analysis, we dichotomized the variable into presence and absence of ED. The internal consistency for this study was good (Cronbach's α = 0.89).

Sleep quality was measured with the Pittsburgh Sleep Quality Index (PSQI). The original scale was designed by Buysse et al. (17) and has nineteen questions grouped in seven components (subjective sleep quality, sleep latency, sleep duration, habitual sleep efficiency, sleep disturbances, use of sleeping medication, and daytime dysfunction), which evaluates sleep quality in the last 4 weeks. The Peruvian version of the PSQI has been validated performing an exploratory factor analysis in a random sample of 4,445 adults over 18 years old from the Estudio Epidemiológico de Lima Metropolitana y Callao, conducted by the Instituto Nacional de Salud Mental in 2012 (18). This version showed an internal consistency of 0.56. Sleep quality was dichotomized into two categories: poor sleep quality (defined as a global PSQI score of 6 or more) and good sleep quality (defined as a global PSQI score of 0 to 5). The internal consistency for this study was acceptable (Cronbach's α = 0.76).

Obstructive sleep apnea was measured with the Berlin questionnaire. The instrument was proposed in 1996 during the Conference on Sleep in Primary Care in Berlin, Germany (19) and has ten questions assessing three categories: snoring, drowsiness, and risk factors. The Berlin questionnaire has been validated (content, criterion, and construct) using a sample of 212 Colombian university students and patients over 18 years old (similar characteristics to the Peruvian population), showing an internal consistency of 0.73 (20). A category was positive if at least two responses indicated a high risk for obstructive sleep apnea. The condition was considered to be present with two or three of the instrument categories were positive. The internal consistency for this study was acceptable (Cronbach's α = 0.74).

Additional sociodemographic data were age (continuous), academic year, and nutritional status (classified as “normal”, “overweight”, and “obesity”, based on self-reported weight and height).

### Statistical analysis

Descriptive data were presented as number (%) for categorical variables and median (min-max values) for non-normally distributed continuous variables. Bivariate differences in the prevalence of ED across covariates were calculated with the chi-square test (for categorical exposures) and Kruskal-Wallis test (for non-normally distributed continuous variables).

For multivariate analysis, prevalence ratios (PR) with 95% confidence intervals (CI) were estimated using generalized linear models with Poisson family distribution, log link function, and robust variance. Using this approach allows (1) to determine the difference between the exposed and unexposed groups for developing ED, (2) to avoid overestimation of the association estimate, and (3) to establish a practical value (PR) that clearly informs policy makers for the formulation of prevention programs. The use of Poisson regression is also convenient because the assumptions are simple compared to linear regression (only the linearity of log(λ) vs. *X* and independence of observations were required). The inclusion of confounding variables for adjusting the regression model was based on epidemiological criteria considering previous literature (21–23) (see Supplementary Figure S1 for the proposed directed acyclic graph). Variance inflation factors (VIF) were also obtained in the regression models to evaluate potential multicollinearity.

Alternative analyses were presented using different scales of measurement for the exposure and outcome. First, we performed a multinomial logistic regression analysis to test the adjusted association between the PSQI score and mild ED versus no ED and mild-to-moderate ED versus no ED. The highest levels of severity were not analyzed because no participant had advanced stages of ED. The multinomial regression coefficient from the model was exponentiated and presented as PR and 95% CI. Interpretation of PR is based originally on the calculation of relative risk ratios, defined as the ratio of the probability of an outcome in the exposed group to the probability of an outcome in the unexposed group (24). Second, to assess the association between sleep quality and erectile dysfunction as continuous variables (using the original scores of PSQI and IIEF-5), locally weighted regression analysis was performed.

A *p*-value <0.05 was considered statistically significant. Analyses were performed in Stata 15.0.

### Ethical considerations

This study was performed in line with the principles of the Declaration of Helsinki. Ethical approval was obtained from the Ethics Committee of the Universidad Ricardo Palma (Lima, Peru). Written informed consent was signed by participants before inclusion in the study. Confidentiality was maintained by using anonymous surveys.

## Results

Of 381 participants, the median age was 23 years (min-max values: 18–29), 32% belonged to the second academic year, 12.3% had obesity, and 13.1% had obstructive sleep apnea. Poor sleep quality was reported by 50.9% of the respondents. Over half of them (54.6%) experienced some degree of ED, 43.3% corresponding to mild ED and 11.3% to mild to moderate ED (Table 1).

**Table 1:** Characteristics of participants (*n* = 381).

**Characteristics**	***n* (%)**
Age (years)^*^	23 (18–29)
**Academic year**	
First	66 (17.3)
Second	122 (32.0)
Third	72 (18.9)
Fourth	121 (31.8)
**Nutritional status**	
Normal	187 (49.1)
Overweight	147 (38.6)
Obesity	47 (12.3)
**Sleep Apnea**	
No	331 (86.9)
Yes	50 (13.1)
**Sleep Quality**	
Good	187 (49.1)
Poor	194 (50.9)
**Erectile dysfunction**	
No	173 (45.4)
Mild	165 (43.3)
Mild to moderate	43 (11.3)

^*^Median (min-max value).

Participants with poor sleep quality had 59.9% higher prevalence of mild ED (72.7 vs. 12.8%, *p* < 0.001) than those with good sleep quality, and 19% higher prevalence of mild to moderate ED than those with good sleep quality (20.6 vs. 1.6%, *p* < 0.001). Also, those in the first academic year had 5.1% higher prevalence of mild ED than those in the fourth year (53 vs. 47.9%, *p* = 0.084), and 2.5% higher prevalence of mild to moderate ED than those in the fourth academic year (9.1 vs. 6.6%, *p* = 0.084). Participants with obesity had 15.2% lower prevalence of mild ED than those with normal BMI (31.9 vs. 47.1%, *p* = 0.234), and 9% higher prevalence of mild to moderate ED than those with normal BMI (19.2 vs. 10.2%, *p* = 0.234). Participants with obstructive sleep apnea had 5.4% higher prevalence of mild to moderate ED than those without obstructive sleep apnea (48 vs. 42.6%, *p* = 0.002), and 14.6% higher prevalence of mild to moderate ED than those without obstructive sleep apnea (24 vs. 9.4%, *p* = 0.002) (Table 2).

**Table 2:** Factors associated with erectile dysfunction in bivariate analysis.

**Variables**	**Erectile dysfunction**			p^**^
	**No** **(*****n** =* **173)**	**Mild** **(*****n** =* **165)**	**Mild to moderate** **(*****n** =* **43)**	
	***n*** **(%)**	***n*** **(%)**		
Age (years)^*^^†^	23 (18 - 29)	23 (18 - 29)	23 (21 - 29)	0.186
**Academic year**				0.084
First	25 (37.9)	35 (53.0)	6 (9.1)	
Second	55 (45.1)	47 (38.5)	20 (16.4)	
Third	38 (52.8)	25 (34.7)	9 (12.5)	
Fourth	55 (45.5)	58 (47.9)	8 (6.6)	
**Nutritional status**				0.234
Normal	80 (42.8)	88 (47.1)	19 (10.2)	
Overweight	70 (47.6)	62 (42.2)	15 (10.2)	
Obesity	23 (48.9)	15 (31.9)	9 (19.2)	
**Sleep apnea**				0.002
No	159 (48.0)	141 (42.6)	31 (9.4)	
Yes	14 (28.0)	24 (48.0)	12 (24.0)	
**Sleep Quality**				<0.001
Good	160 (85.6)	24 (12.8)	3 (1.6)	
Poor	13 (6.7)	141 (72.7)	40 (20.6)	

^*^Median (min-max value).

^†^P-value calculated with the Kruskal-Wallis's test.

^**^P-values calculated with the chi-square test.

**Figure 1:**
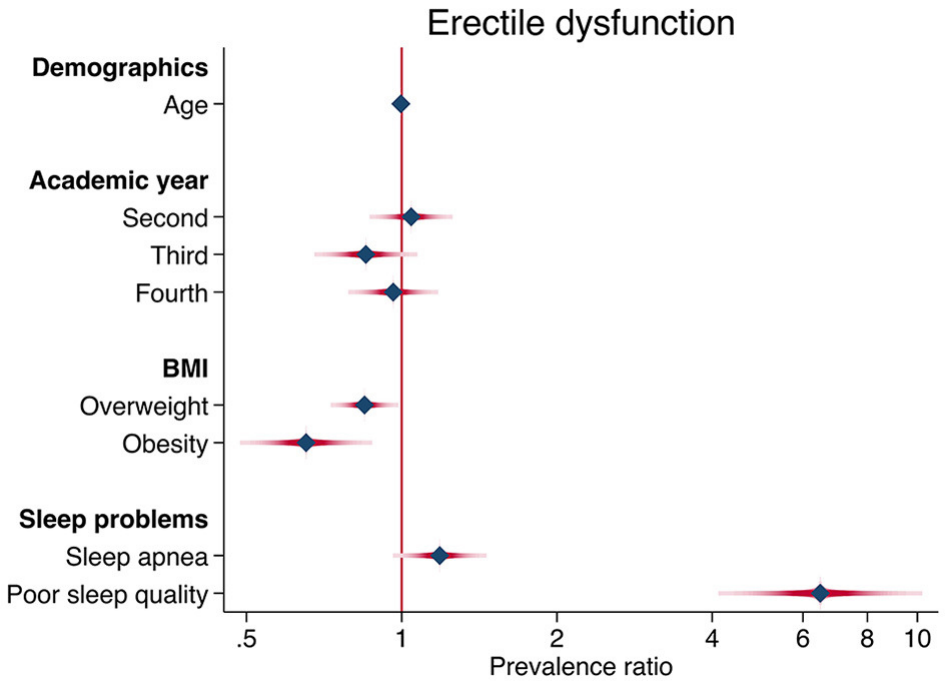
Forest plot of the factors associated with erectile dysfunction. Prevalence ratios are represented by blue diamond symbols and confidence intervals by red horizontal lines.

In the simple regression model (Table 3), poor sleep quality was significantly associated with a higher prevalence of ED (PR=6.46; 95% CI: 4.55–9.18). After adjusting for age, academic year, nutritional status, and obstructive sleep apnea, this association remained constant (aPR = 6.48; 95% CI: 4.58–9.17). A graphical representation of the multivariate analysis is shown in Figure 1.

**Table 3:** Association between sleep quality and erectile dysfunction in simple and multiple regression analysis.

**Characteristics**	**Simple Regression**			**Multiple Regression** ^**^		
	**PR**	**95% CI**	**p** ^*^	**PR**	**95% CI**	**p** ^*^
Age (years)	1.01	0.98–1.05	0.412	1.00	0.97–1.02	0.745
**Academic year**						
First	Ref.			Ref.		
Second	0.88	0.69–1.13	0.330	1.04	0.91–1.20	0.559
Third	0.76	0.56–1.04	0.082	0.85	0.72–1.02	0.073
Fourth	0.88	0.68–1.13	0.306	0.96	0.83–1.12	0.629
**Nutritional status**						
Normal	Ref.			Ref.		
Overweight	0.92	0.75–1.12	0.382	0.85	0.76–0.95	0.005
Obesity	0.89	0.66–1.21	0.467	0.65	0.52–0.82	<0.001
**Sleep apnea**						
No	Ref.			Ref.		
Yes	1.39	1.13–1.70	0.002	1.19	1.01–1.39	0.036
**Sleep quality**						
Good	Ref.			Ref.		
Poor	6.46	4.55–9.18	<0.001	6.48	4.58–9.17	<0.001

^*^P-values calculated with generalized linear models with Poisson family, log link function, and robust variance.

^**^Adjusted for age, academic year, nutritional status, and sleep apnea.

Results from the multinomial logistic regression analysis (Supplementary Table S1) showed that the PR of having mild ED over not having ED was 3.04 per unit increase in PSQI global score, and the PR of having mild-to-moderate ED over not having ED was 4.61 per unit increase in PSQI global score. In addition, locally weighted regression analysis showed a negative relationship between PSQI and IIEF-5 scores (Supplementary Figure S2).

## Discussion

### Main findings

The prevalence of ED was present in over half of the participants and mild ED was the most common form of severity. This study also evidenced that poor sleep quality was independently associated with a higher prevalence of ED.

### Plausibility of findings

The results support the hypothesis that a considerable number of young university students suffer from ED. This is in line with previous literature stating that ED is an important but underreported feature among students (25). The most feasible explanation is the presence of stressors triggering psychological ED (e.g., somatization, interpersonal sensitivity, and depression) (26), which is relevant in this life stage due to academic exigence and potential uncertainty in life.

In addition, the study supports the hypothesis that poor sleep quality affects erectile function. It has been reported that insomnia or insufficient sleep can shorten testosterone levels (27, 28). Since sleep duration is commonly affected during university stage, this phenomenon may contribute considerably to the reduction of testosterone levels and therefore the sexual capacity in male students.

It is possible that sleep quality mediates the effect of psychological status on ED. Mental disorders may also confound the association between poor sleep quality and ED (29). Since we did not measure potential psychogenic factors for ED (e.g., distress, anxiety, depression), the association identified in this study could be overestimated.

### Comparison with previous studies

The prevalence of ED found in this study is higher than that reported in the literature. A study in Peru showed a lower estimate among medical students (28% with mild symptoms) (13). Another multinational study reported an ED prevalence of 8% among men aged 20–29 years (30). In Brazil, a population-based survey identified ED in 7% of men aged 20–29 years (31), and another study in this country showed a frequency of 35% (32). In Israel, mild ED was present in 22% of military members aged under 40 years (33). Methodological differences could mainly explain these variations, but also the age group, type of activity, and cultural aspects. Despite of this, there is a trend showing that sexual dysfunction arises as a common problem among young men (30). Therefore, preventive measures should be established among university students in order to cope with emotional problems during this stage (29, 34–36).

This study showed that 93.3% of students with poor sleep quality experienced some form of ED. Furthermore, poor sleep quality increased significantly the prevalence of ED (over 600%). These findings add to previous estimates of sleep problems in men with ED. For example, a cohort study in Taiwan showed that 60% of men with ED (aged 20–39 years) suffered from sleep apnea and 43% from sleep disorders (37). Other studies have demonstrated that poor sleep quality is a risk factor for ED in young adults (38–40). In a Taiwanese study, the incidence of erectile dysfunction was three times higher in men with sleep disorders than in men without this condition (11). In the Peruvian context, a study in medical students showed a significant association between poor sleep quality and ED, although confounding variables were not included in the analysis (13).

This study found in the exploratory analysis that sleep apnea was associated with a 19% higher prevalence of ED. There are many studies supporting the association between sleep apnea and ED (11, 38, 39, 41, 42). For example, it was shown that the risk of ED was ten times higher in men with sleep apnea than in men without this condition (37). In addition, a meta-analysis reported a 55% lower risk of ED in men without obstructive sleep apnea (43). However, this association was shown to occur only in men over 65 years of age, a finding attributed to the mediation of age-related oxygen desaturation (44).

Although the most common cause of sleep apnea in young people is excess weight (6), an independent association between sleep apnea and ED was found after controlling for nutritional status. This result may be explained by the presence of a psychological factor impairing the normal breathing pattern during sleep (6). However, the effect of hypoxemia on erectile dysfunction has been extensively described (6, 44), suggesting that sleep apnea contributes independently to the presence of erectile dysfunction.

This study also showed that impaired nutritional status was associated with a lower prevalence of ED (15% lower for overweight and 35% lower for obesity). This is contrary to what is commonly expected since weight gain could affect endothelial function through altered metabolic activity and serum testosterone, considered to be the main mechanisms of ED (12). Although some reports have identified an increased risk of ED in patients with obesity, others did not find any significant association (12, 32, 45). The result found in this study may be caused by statistical confusion of unmeasured psychological factors, such as depression and anxiety. Another reason may be that students did not face overweight or obesity as chronic conditions, and that have not yet affected their erectile function. A third reason could be that the number of participants from the groups of obesity and overweight were too small to significantly differentiate the prevalence of ED.

### Limitations and strengths

The study had several limitations. First, its cross-sectional design did not allow to infer causality because variables were measured at the same time. Second, the study collected self-reported data, introducing information bias that could have modified the association estimate. This is particularly important for self-reported weight and height since Peruvian and international studies has shown that calculation of BMI from this type of measure may be altered by sex and aging (46, 47). Third, the sampling method was non-probabilistic, potentially leading to an inaccurate prevalence of ED. Therefore, the results should be interpreted with caution due to limited internal/external validity and reliability. Despite these limitations, the results are supported by validated instruments and acceptable sample size. Furthermore, the study addressed a neglected topic in young university students, which may reinforce the importance of sleep hygiene and encourage the design of more robust research.

### Recommendations for future research

ED can cause anxiety in young men due to lower perception of masculinity and sexuality. The development of psychological disorders may also reinforce the severity of erectile dysfunction due to altered sexual arousal (48). These assumptions are supported by several population-based studies (29, 34–37, 49). However, only 58% of patients with erectile dysfunction seek medical care (30), and one out of four patients is under 40 years of age (25). Therefore, it is essential to establish preventive and intervention programs in mental health to reduce the risk of ED in young people. For this purpose, a more robust design and a representative sample of Peruvian students should be included in future studies. Also, psychological status should be measured and included in the analysis as a potential confounding variable.

### Proposed support structures for universities

Student welfare and academic achievement are essential objectives for universities. To help reduce the risk of developing erectile dysfunction, universities should create screening programs that assess the psychological state of male students, recognizing that there are certain personality types that might increase this risk (50). Since sleep problems are part of emotional disorders (10), it would be useful to have ongoing psychological assessment to identify which stressful periods trigger this condition (e.g., exam periods). It would also be important to investigate the quality of relationships with other students, peers, teachers, and family. Consideration should be given to promoting sleep hygiene and avoiding excessive study hours and overnight stays. In addition, medical check-ups should be carried out to evaluate the sexual health of students, in which erectile dysfunction can be recognized early and treated if necessary. Since the literature is still scarce on ED among young male students, universities should promote research on this topic and identify the prevalence and possible risk factors for ED.

## Conclusion

Poor sleep quality was independently associated with ED. This finding suggests that male students are at risk for sexual problems due to possible academic demands and relationship issues. This result was also supported by the high prevalence of mild and mild to moderate ED found in this group of students. Future research should address common student characteristics that may increase the risk of sleep problems. In this way, better preventive programs could be established in universities.

## Data availability statement

The raw data supporting the conclusions of this article will be made available by the authors, without undue reservation.

## Ethics statement

The studies involving human participants were reviewed and approved by Ethics Committee of the Universidad Ricardo Palma (Lima, Peru). The patients/participants provided their written informed consent to participate in this study.

## Author contributions

PG-V: conception and design of the work, acquisition, analysis, and interpretation of data, drafted the work and revised it critically, and approved the version to be published. MV-G: analysis and interpretation of data, revised the work critically, and approved the version to be published. CP and VV-P: interpretation of data, drafted the work and revised it critically, and approved the version to be published. JG-U: design of the work, analysis and interpretation of data, revised the work critically, and approved the version to be published. All authors contributed to the article and approved the submitted version.

## References

[1] Feldman HA Goldstein I Hatzichristou DG Krane RJ McKinlay JB. Impotence and its medical and psychosocial correlates: results of the Massachusetts Male Aging Study. J Urol. (1994) 151:54–61. 10.1016/S0022-5347(17)34871-1 8254833

[2] Goldstein I Goren A Li VW Tang WY Hassan TA. Epidemiology update of erectile dysfunction in eight countries with high burden. Sex Med Rev. (2020) 8:48–58. 10.1016/j.sxmr.2019.06.008 31416758

[3] Santibáñez C Anchique C Herdy A Zeballos C González G Fernández R. Prevalencia de disfunción eréctil y factores asociados en pacientes con indicación de rehabilitación cardíaca. Revista chilena de cardiologí*a*. (2016) 35:216–21. 10.4067/S0718-85602016000300002

[4] Rastrelli G Maggi M. Erectile dysfunction in fit and healthy young men: psychological or pathological? Transl Androl Urol. (2017) 6:79–90. 10.21037/tau.2016.09.06 28217453 PMC5313296

[5] Jern P Gunst A Sandnabba K Santtila P. Are early and current erectile problems associated with anxiety and depression in young men? A retrospective self-report study. J Sex Marital Ther. (2012) 38:349–64. 10.1080/0092623X.2012.665818 22712819

[6] Cho JW Duffy JF. Sleep, sleep disorders, and sexual dysfunction. World J Mens Health. (2019) 37:261–75. 10.5534/wjmh.180045 30209897 PMC6704301

[7] Chaput JP Dutil C Featherstone R Ross R Giangregorio L Saunders TJ. Sleep duration and health in adults: an overview of systematic reviews. Appl Physiol Nutr Metab. (2020) 45:S218–31. 10.1139/apnm-2020-0034 33054337

[8] Itani O Jike M Watanabe N Kaneita Y. Short sleep duration and health outcomes: a systematic review, meta-analysis, and meta-regression. Sleep Med. (2017) 32:246–56. 10.1016/j.sleep.2016.08.006 27743803

[9] Pavlova MK Latreille V. Sleep disorders. Am J Med. (2019) 132:292–9. 10.1016/j.amjmed.2018.09.021 30292731

[10] Scott J Kallestad H Vedaa O Sivertsen B Etain B. Sleep disturbances and first onset of major mental disorders in adolescence and early adulthood: A systematic review and meta-analysis. Sleep Med Rev. (2021) 57:101429. 10.1016/j.smrv.2021.101429 33549912

[11] Lin HH Ho FM Chen YF Tseng CM Ho CC Chung WS. Increased risk of erectile dysfunction among patients with sleep disorders: A nationwide population-based cohort study. Int J Clin Pract. (2015) 69:846–52. 10.1111/ijcp.12629 25708176

[12] Pizzol D Smith L Fontana L Caruso MG Bertoldo A Demurtas J. Associations between body mass index, waist circumference and erectile dysfunction: A systematic review and meta-analysis. Rev Endocr Metab Disord. (2020) 21:657–66. 10.1007/s11154-020-09541-0 32002782

[13] Grandez-Urbina LA Montealegre-Inuma J Galindo-Huamani Z Correa-Lopez L Helguero-Santin LM Pichardo-Rodriguez R. Erectile dysfunction associated to decrease in sleep quality in young adults of a Peruvian university. J Sex Med. (2018) 15:S171. 10.1016/j.jsxm.2018.04.106

[14] Rosen R Cappelleri J Smith M Lipsky J Peña B. Constructing and evaluating the “Sexual Health Inventory for Men: IIEF-5” as a diagnostic tool for erectile dysfunction. Int J Impot Res. (1998) 10:S35.

[15] Zegarra L Loza C Pérez V. Validación psicométrica del instrumento índice internacional de función eréctil en pacientes con disfunción eréctil en Perú. Rev Peru Med Exp Salud Publica. (2011) 28:477–83. 10.1590/S1726-46342011000300011 22086628

[16] van Kollenburg RAA de Bruin DM Wijkstra H. Validation of the electronic version of the International Index of Erectile Function (IIEF-5 and IIEF-15): A crossover study. J Med Internet Res. (2019) 21:e13490. 10.2196/13490 31267983 PMC6634948

[17] Buysse DJ Reynolds CF Monk TH Berman SR Kupfer DJ. The Pittsburgh sleep quality index: a new instrument for psychiatric practice and research. Psychiatry Res. (1989) 28:193–213. 10.1016/0165-1781(89)90047-4 2748771

[18] Luna-Solis Y Robles-Arana Y Aguero-Palacios Y. Validarion of the Pittsburgh sleep quality index in a peruvian sample. Anales de salud mental. (2015) 21:23–30. Available online at: https://www.academia.edu/34647099/VALIDACIÓN_DEL_ÍNDICE_DE_CALIDAD_DE_SUEÑO_DE_PITTSBURGH_EN_UNA_MUESTRA_PERUANA_VALIDATION_OF_THE_PITTSBURGH_SLEEP_QUALITY_INDEX_IN_A_PERUVIAN_SAMPLE

[19] Netzer NC Stoohs RA Netzer CM Clark K Strohl KP. Using the Berlin Questionnaire to identify patients at risk for the sleep apnea syndrome. Ann Intern Med. (1999) 131:485–91. 10.7326/0003-4819-131-7-199910050-00002 10507956

[20] Polanía-Dussan IG Escobar-Córdoba F Eslava-Schmalbach J Netzer NC. Validación colombiana del cuestionario de Berlín. Revista de la Facultad de Medicina. (2013) 61:8.

[21] Rodriguez KM Kohn TP Kohn JR Sigalos JT Kirby EW Pickett SM. Shift work sleep disorder and night shift work significantly impair erectile function. J Sex Med. (2020) 17:1687–93. 10.1016/j.jsxm.2020.06.009 32736945 PMC7484090

[22] Nyer M Farabaugh A Fehling K Soskin D Holt D Papakostas GI. Relationship between sleep disturbance and depression, anxiety, and functioning in college students. Depress Anxiety. (2013) 30:873–80. 10.1002/da.22064 23681944 PMC3791314

[23] Budweiser S Enderlein S Jörres RA Hitzl AP Wieland WF Pfeifer M. Sleep apnea is an independent correlate of erectile and sexual dysfunction. J Sex Med. (2009) 6:3147–57. 10.1111/j.1743-6109.2009.01372.x 19570042

[24] Camey SA Torman VBL Hirakata VN Cortes RX Vigo A. Bias of using odds ratio estimates in multinomial logistic regressions to estimate relative risk or prevalence ratio and alternatives. Cad Saude Publica. (2014) 30:21–9. 10.1590/0102-311X00077313 24627010

[25] Yafi FA Jenkins L Albersen M Corona G Isidori AM Goldfarb S. Erectile dysfunction. Nat Rev Dis Primers. (2016) 2:16003. 10.1038/nrdp.2016.3 27188339 PMC5027992

[26] Aghighi A Grigoryan VH Delavar A. Psychological determinants of erectile dysfunction among middle-aged men. Int J Impot Res. (2015) 27:63–8. 10.1038/ijir.2014.34 25164317

[27] Luboshitzky R Zabari Z Shen-Orr Z Herer P Lavie P. Disruption of the nocturnal testosterone rhythm by sleep fragmentation in normal men. J Clin Endocrinol Metab. (2001) 86:1134–9. 10.1210/jcem.86.3.7296 11238497

[28] Schmid SM Hallschmid M Jauch-Chara K Lehnert H Schultes B. Sleep timing may modulate the effect of sleep loss on testosterone. Clin Endocrinol (Oxf). (2012) 77:749–54. 10.1111/j.1365-2265.2012.04419.x 22568763

[29] Cheng QS Liu T Huang HB Peng YF Jiang SC Mei XB. Association between personal basic information, sleep quality, mental disorders and erectile function: A cross-sectional study among 334 Chinese outpatients. Andrologia. (2017) 49:570–5. 10.1111/and.12631 27364774

[30] Rosen RC Fisher WA Eardley I Niederberger C Nadel A Sand M. The multinational Men's Attitudes to Life Events and Sexuality (MALES) study: Prevalence of erectile dysfunction and related health concerns in the general population. Curr Med Res Opin. (2004) 20:607–17. 10.1185/030079904125003467 15171225

[31] Andersen ML Santos-Silva R Bittencourt LRA Tufik S. Prevalence of erectile dysfunction complaints associated with sleep disturbances in São Paulo, Brazil: A population-based survey. Sleep Med. (2010) 11:1019–24. 10.1016/j.sleep.2009.08.016 20427234

[32] Martins FG Abdo CHN. Erectile dysfunction and correlated factors in Brazilian men aged 18-40 years. J Sex Med. (2010) 7:2166–73. 10.1111/j.1743-6109.2009.01542.x 19889149

[33] Heruti R Shochat T Tekes-Manova D Ashkenazi I Justo D. Prevalence of erectile dysfunction among young adults: Results of a large-scale survey. J Sex Med. (2004) 1:284–91. 10.1111/j.1743-6109.04041.x 16422958

[34] Goldstein I Chambers R Tang W Stecher V Hassan T. Real-world observational results from a database of 48 million men in the United States: Relationship of cardiovascular disease, diabetes mellitus and depression with age and erectile dysfunction. Int J Clin Pract. (2018) 72:e13078. 10.1111/ijcp.13078 29569323

[35] Yang Y Song Y Lu Y Xu Y Liu L Liu X. Associations between erectile dysfunction and psychological disorders (depression and anxiety): A cross-sectional study in a Chinese population. Andrologia. (2019) 51:e13395. 10.1111/and.13395 31434163

[36] Zhang K He LJ Yu W Wang Y Bai WJ Wang XF. Association of depression/anxiety with lower urinary tract symptoms and erectile dysfunction in Chinese men aged from 22 to 50 years. Beijing Da Xue Xue Bao Yi Xue Ban. (2013) 45:609–12. Available online at: http://xuebao.bjmu.edu.cn/EN/Y2013/V45/I4/609 23939173

[37] Chen KF Liang SJ Lin CL Liao WC Kao CH. Sleep disorders increase risk of subsequent erectile dysfunction in individuals without sleep apnea: A nationwide population-base cohort study. Sleep Med. (2016) 17:64–8. 10.1016/j.sleep.2015.05.018 26847976

[38] Chen CM Tsai MJ Wei PJ Su YC Yang CJ Wu MN. Erectile dysfunction in patients with sleep apnea–A nationwide population-based study. PLoS ONE. (2015) 10:e0132510. 10.1371/journal.pone.0132510 26177206 PMC4503619

[39] Hirshkowitz M Karacan I Arcasoy MO Acik G Narter EM Williams RL. Prevalence of sleep apnea in men with erectile dysfunction. Urology. (1990) 36:232–4. 10.1016/0090-4295(90)80262-L 2392814

[40] Seehuus M Pigeon W. The sleep and sex survey: Relationships between sexual function and sleep. J Psychosom Res. (2018) 112:59–65. 10.1016/j.jpsychores.2018.07.005 30097137

[41] Kalejaiye O Raheem AA Moubasher A Capece M McNeillis S Muneer A. Sleep disorders in patients with erectile dysfunction. BJU Int. (2017) 120:855–60. 10.1111/bju.13961 28710780

[42] Teloken PE Smith EB Lodowsky C Freedom T Mulhall JP. Defining association between sleep apnea syndrome and erectile dysfunction. Urology. (2006) 67:1033–7. 10.1016/j.urology.2005.11.040 16698364

[43] Kellesarian SV Malignaggi VR Feng C Javed F. Association between obstructive sleep apnea and erectile dysfunction: A systematic review and meta-analysis. Int J Impot Res. (2018) 30:129–40. 10.1038/s41443-018-0017-7 29795528

[44] Martin SA Appleton SL Adams RJ Taylor AW Vincent A Brook NR. Erectile dysfunction is independently associated with apnea-hypopnea index and oxygen desaturation index in elderly, but not younger, community-dwelling men. Sleep Health. (2017) 3:250–6. 10.1016/j.sleh.2017.04.006 28709511

[45] Moon KH Park SY Kim YW. Obesity and erectile dysfunction: From bench to clinical implication. World J Mens Health. (2019) 37:138–47. 10.5534/wjmh.180026 30079640 PMC6479091

[46] Loret de. Mola C, Pillay TD, Diez-Canseco F, Gilman RH, Smeeth L, Miranda JJ. Body Mass Index and Self-Perception of Overweight and Obesity in Rural, Urban and Rural-to-Urban Migrants: PERU MIGRANT Study. PLoS ONE. (2012) 7:e50252. 10.1371/journal.pone.0050252 23209688 PMC3508895

[47] Merrill RM Richardson JS. Validity of self-reported height, weight, and body mass index: findings from the national health and nutrition examination survey, 2001-2006. Prev Chronic Dis. (2009) 6:A121. 19754997 PMC2774635

[48] Hale VE Strassberg DS. The role of anxiety on sexual arousal. Arch Sex Behav. (1990) 19:569–81. 10.1007/BF01542466 2082861

[49] Mialon A Berchtold A Michaud PA Gmel G Suris JC. Sexual dysfunctions among young men: Prevalence and associated factors. J Adolesc Health. (2012) 51:25–31. 10.1016/j.jadohealth.2012.01.008 22727073

[50] Fan YH Liou YJ Cheng WM. Type D Personality independently predicts erectile dysfunction in taiwanese young men. J Sex Med. (2022) 19:1397–403. 10.1016/j.jsxm.2022.06.012 35882608

