# Supplementary figures and images for: Poor sleep quality and erectile dysfunction in students from a Peruvian University: A cross-sectional study
Source: Front Public Health. 2023 Feb 1;11:932718. doi: 10.3389/fpubh.2023.932718 (PMC9928877; doi:10.3389/fpubh.2023.932718)

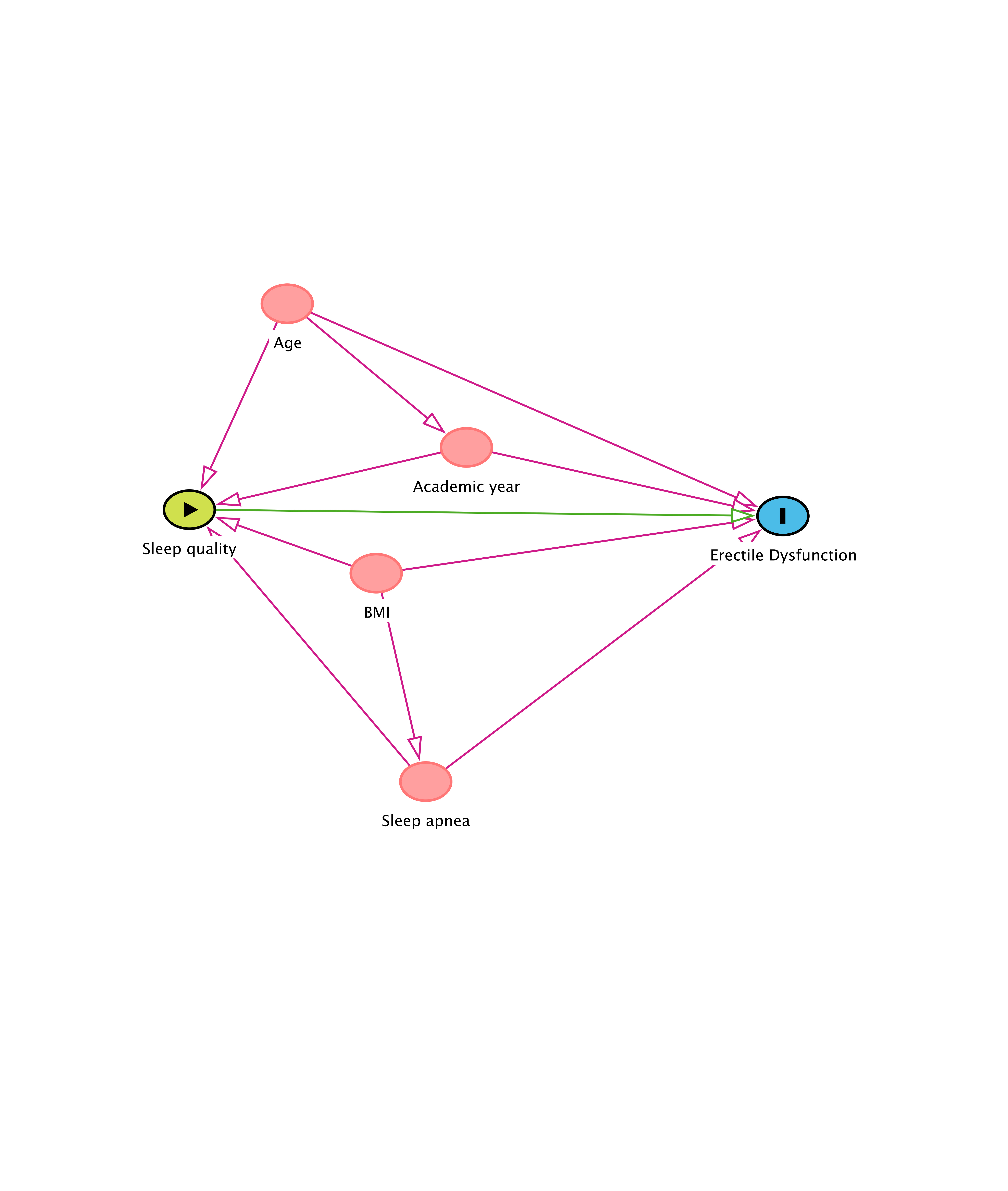

Supplement: Supplementary file 1 [file Image_1.TIF]

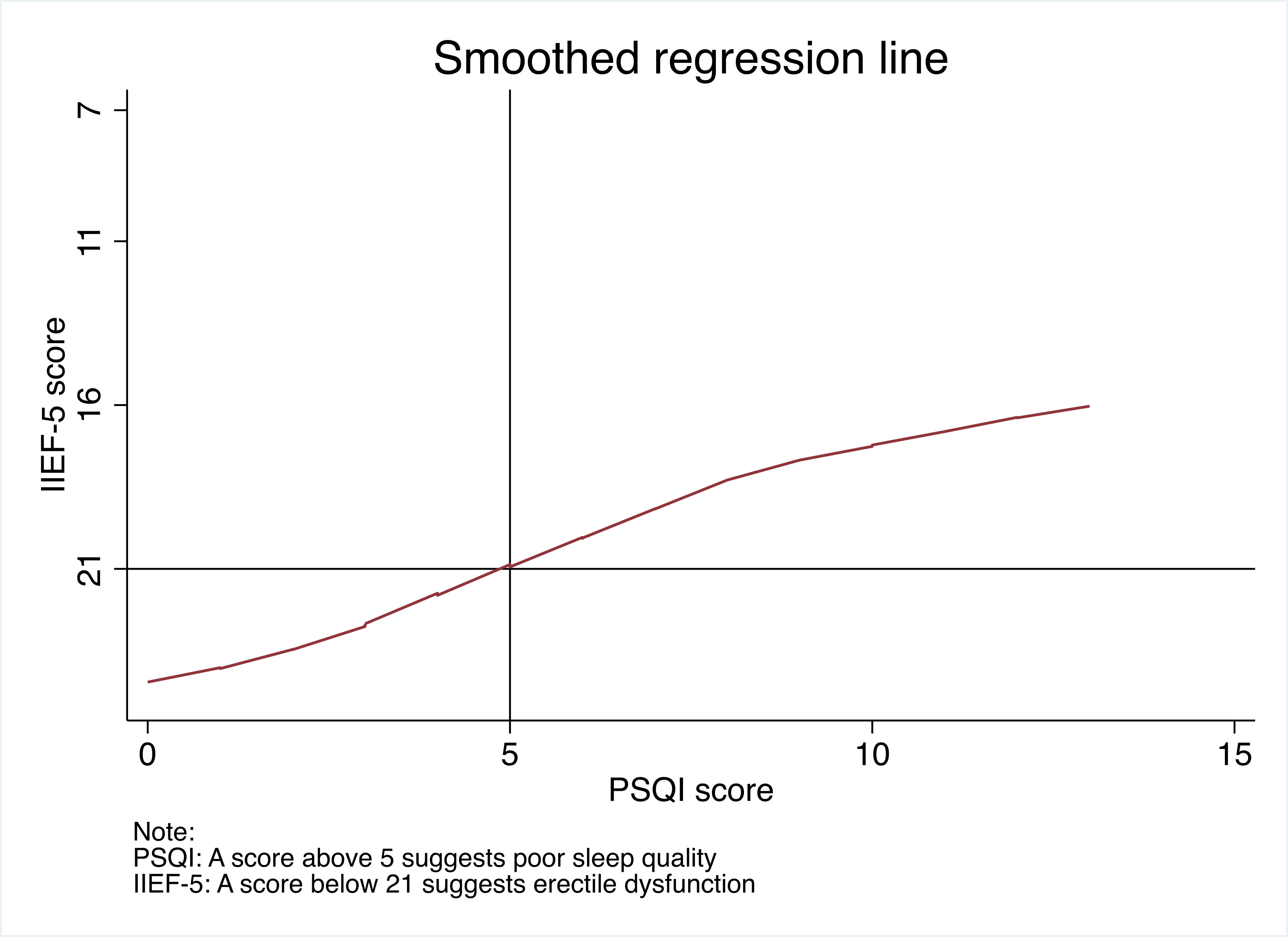

Supplement: Supplementary file 2 [file Image_2.TIF]
